# Uncoupling of Vascular Nitric Oxide Synthase Caused by Intermittent Hypoxia

**DOI:** 10.1155/2016/2354870

**Published:** 2016-10-20

**Authors:** Mohammad Badran, Bisher Abuyassin, Saeid Golbidi, Najib Ayas, Ismail Laher

**Affiliations:** ^1^Department of Anesthesiology, Pharmacology and Therapeutics, University of British Columbia, Vancouver, BC, Canada; ^2^Divisions of Critical Care and Respiratory Medicine, Department of Medicine, University of British Columbia, Vancouver, BC, Canada; ^3^Sleep Disorders Program, UBC Hospital, Vancouver, BC, Canada; ^4^Division of Critical Care Medicine, Providence Healthcare, Vancouver, BC, Canada

## Abstract

*Objective*. Obstructive sleep apnea (OSA), characterized by chronic intermittent hypoxia (CIH), is often present in diabetic (DB) patients. Both conditions are associated with endothelial dysfunction and cardiovascular disease. We hypothesized that diabetic endothelial dysfunction is further compromised by CIH.* Methods*. Adult male diabetic (BKS.Cg-*Dock7* ^*m*^ +/+* Lepr* ^*db*^/J) (*db/db*) mice (10 weeks old) and their heterozygote littermates were subjected to CIH or intermittent air (IA) for 8 weeks. Mice were separated into 4 groups: IA (intermittent air nondiabetic), IH (intermittent hypoxia nondiabetic), IADB (intermittent air diabetic), and IHDB (intermittent hypoxia diabetic) groups. Endothelium-dependent and endothelium-independent relaxation and modulation by basal nitric oxide (NO) were analyzed using wire myograph. Plasma 8-isoprostane, interleukin-6 (IL-6), and asymmetric dimethylarginine (ADMA) were measured using ELISA. Uncoupling of eNOS was measured using dihydroethidium (DHE) staining.* Results*. Endothelium-dependent vasodilation and basal NO production were significantly impaired in the IH and IADB group compared to IA group but was more pronounced in IHDB group. Levels of 8-isoprostane, IL-6, ADMA, and eNOS uncoupling were ≈2-fold higher in IH and IADB groups and were further increased in the IHDB group.* Conclusion*. Endothelial dysfunction is more pronounced in diabetic mice subjected to CIH compared to diabetic or CIH mice alone. Oxidative stress, ADMA, and eNOS uncoupling were exacerbated by CIH in diabetic mice.

## 1. Introduction

Obstructive sleep apnea (OSA) involves obstruction of the airways for at least 10 seconds (apnea) or sharp decreases in breathing amplitude (hypopnea) during sleep [1]. OSA is an independent risk factor for cardiovascular disease (CVD) [2] and is also associated with other CVD risk factors such as obesity [3, 4], diabetes [5, 6], and hypertension [7]. The cardiovascular pathology of OSA has been primarily linked to chronic intermittent hypoxia (CIH) and increased sympathetic innervation [8]. OSA leads to CVD through several pathological mechanisms if left untreated [9]; an important mechanism is through vascular endothelial dysfunction characterized by impaired nitric oxide (NO) production, which can ultimately lead to atherosclerosis [10]. Numerous clinical and animal studies demonstrate that OSA and CIH lead to endothelial dysfunction [11–14].

There is much support for a bidirectional association between OSA and diabetes [5]. The estimated prevalence of OSA in diabetic patients is approximately 71%, and 15%–30% of patients with OSA have diabetes [15]. The risk of developing diabetes is related to the severity of OSA [16]. Diabetes is associated with cardiovascular disease and increased mortality [17, 18], where nearly 70% of people aged 65 or older with diabetes die from heart disease and are 2 to 4 times more likely to suffer from heart disease and stroke [17]. Endothelial dysfunction is a hallmark of diabetes, with oxidative stress and inflammation having important roles in the process [1]. We hypothesized that CIH further exacerbates endothelial dysfunction of diabetic mice.

## 2. Materials and Methods

### 2.1. Animal Groups

Experimental protocols were approved by the Animal Care Center at the University of British Columbia, Canada (certificate number A15-0146). Adult male (10 wks. old) BKS.Cg-*Dock7* ^*m*^ +/+* Lepr* ^*db*^/J (*db/db* –* Lepr* ^*db*^) mice and their heterozygote littermates (*Lepr* ^*db*^ */+*) were purchased from Jackson Laboratory (Bar Harbour, ME) and housed in the University Animal Resource Unit with 12:12 hours' light/dark cycle with free access to regular mouse chow and water. Unlike their littermates, heterozygotes (*Lepr* ^*db*^ */+*) have normal body weights plasma glucose, insulin, and leptin levels. Mice were divided into four groups: (1)* Lepr* ^*db*^ */+* intermittent air nondiabetic (IA), (2)* Lepr* ^*db*^ */+* chronic intermittent hypoxia nondiabetic (IH), (3)* db/db* intermittent air diabetic (IADB), and (4)* db/db* chronic intermittent hypoxia diabetic (IHDB).

### 2.2. CIH Protocol

We used a validated rodent model as described previously [11]. Mice were housed in customized cages with ports evenly spaced near the bottom of the cages to allow gas to enter from all sides. A gas control delivery system regulates the flow of compressed air and N_2_ into the cages. Programmable solenoids and flow regulators control the adjustment of inspired O_2_ fraction (FIO_2_) levels in each cage over a wide range of IH profiles. During the 12 hours of light cycle (when mice are sleeping), FIO_2_ was reduced from 21% to 6-7% over a 30-second period and rapidly restored to 21% using a burst of air from a medical air compressor during the following 30 seconds, for a total of 60 cycles per hour for 8 weeks. Room air was delivered to the cages throughout the 12 hours of the dark cycle. For the IA protocol, mice received intermittent air for 12 hours in the light cycle (no N_2_), followed by constant air for the 12 hours of the dark cycle. The use of multiple inputs into the cage produced a uniform nadir FIO_2_ level throughout the cage. The fluctuating FIO_2_ levels were monitored with an O_2_ analyzer. The nadir FIO_2_ was initially set to 18% and then gradually reduced to 14%, 10%, and 8% and then to the experimental level of nadir FIO_2_ of 6-7% to allow the mice to acclimate to the hypoxic conditions. The oxyhemoglobin saturation reached 55–60% in mice at FIO_2_ of 6-7%.

### 2.3. Plasma and Tissue Collection

Mice were anesthetized with pentobarbital (100 mg/kg) and then euthanized by removing the heart after blood collection. Blood samples were withdrawn from the inferior vena cava by means of heparinized syringes for plasma separation. Plasma was collected after centrifugation (10 mins, 1000 ×g at 4°C) and kept at −80°C. Aortic blood vessels were excised and immersed in chilled oxygenated physiologic salt solution (PSS). Some segments were prepared for functional studies (isometric force recordings) while other aortic segments were frozen in optimal cutting temperature compound (OCT compound, Tissue Tek) and then cryosectioned for dihydroethidium (DHE) staining.

### 2.4. Vascular Endothelial Function

Cleaned aortic blood vessels were cut into equal 2 mm rings and mounted on a wire myograph for measuring isometric tension (DMT 620M, Danish Myotechnology, Aarhus, Denmark) as described previously [18]. Each myograph chamber contained PSS kept at 37°C and pH 7.4 with constant administration of 95% O_2_ and 5% CO_2_ gases. Blood vessels were stretched to their optimal tension (5.5 mN) and allowed to equilibrate for 20 mins before being challenged with 80 mM KCl and then rested in normal PSS again.

For endothelium-dependent and endothelium-independent vasodilation, aortic rings were preconstricted with a submaximal dose of phenylephrine (PE, 1 *μ*M) and followed by cumulative additions of half-log concentrations (10^−10^–10^−5^ M) of acetylcholine (ACh) for endothelium-dependent relaxation; this was repeated with sodium nitroprusside (SNP 10^−10^–10^−5^ M) for endothelium-independent relaxation. For determining the role of basal NO production, two consecutive PE concentration response curves were constructed in the absence and the presence of N_*ω*_-nitro-l-arginine methyl ester hydrochloride (L-NAME, 10^−4^ M). L-NAME inhibits eNOS to reduce intrinsic (basal) NO production, so causing a greater increase in PE-induced vasoconstriction in proportion to the extent of basal NO produced. Basal NO production is estimated by the difference between the two PE curves and measured by the area under the curve (AUC) as we described elsewhere [19, 20].

### 2.5. Biochemical Measures

Plasma 8-isoprostane (indicator of oxidative stress) and interleukin-6 (IL-6, indicator of inflammation) were measured using enzyme-linked immunoassay (ELISA) (Cayman Chemical, Ann Arbor, MI) as was plasma asymmetric dimethylarginine (ADMA) (Eaglebio, Nashua, NH). Some plasma samples were diluted to be within the standard curve as described by the manufacturer.

### 2.6. DHE Staining for eNOS Uncoupling

For identifying eNOS as a source of superoxide, aortic rings were incubated in L-NAME (500 *μ*M) for 30 mins at 37°C before freezing for cryosectioning as previously described [21]. Cryosections were incubated with the superoxide-sensitive fluorescent dye dihydroethidium (DHE (1 *μ*M), Molecular Probes) in a humidity chamber for 30 mins at 37°C. Cover slips were then placed on the slides and kept in the dark for 20 mins. Fluorescence was detected (absorbance: 518 nm, emission: 605 nm) using Olympus BX61 microscope with a RetigaEXi camera (QImaging, Surrey, Canada) and images were analyzed using corrected total cell fluorescence (CTCF) method using ImageJ software (NIH, Bethesda, MD).

### 2.7. Statistical Analysis

All values are expressed as means ± SD. Vascular function data were recorded and analyzed by Powerlab 4/25 and Labchart 7 reader (AD instruments, Australia). Two-way ANOVA with multiple comparisons followed by Bonferroni post hoc test was used to assess differences in the 4 groups; unpaired Student's *t*-tests were used for within-group analysis of tissues before and after L-NAME in the DHE staining experiments using Prism version 6.0 (GraphPad software, California, USA).

## 3. Results

### 3.1. ACh-Induced Endothelial Relaxation

**Figure 1:**
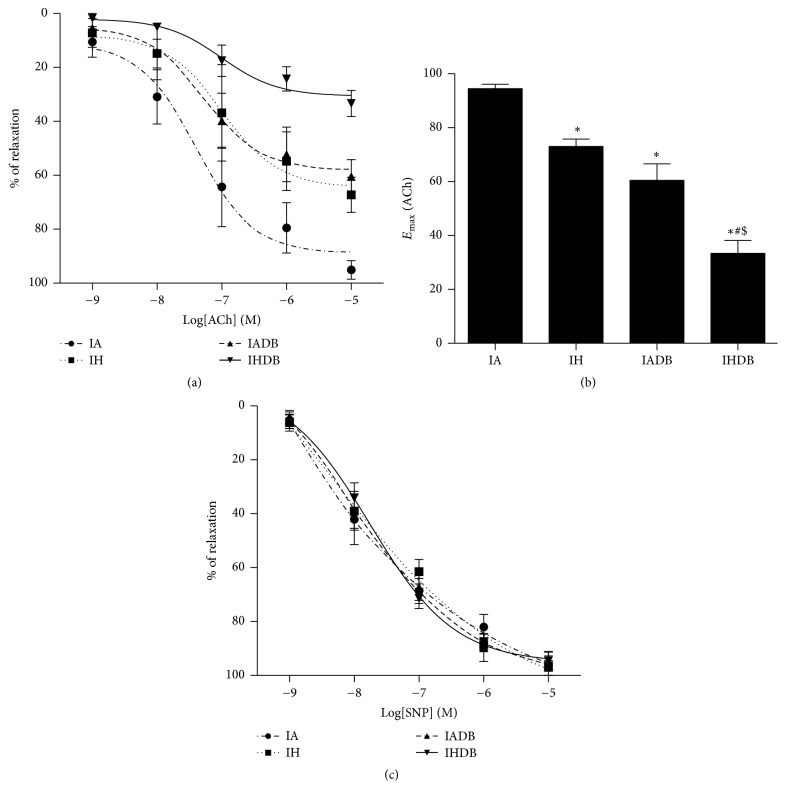
Endothelium-dependent and endothelium-independent relaxation responses in diabetic and nondiabetic mice aortic tissues after 8 weeks of CIH or IA. Cumulative concentration response curve to ACh (a) and maximum relaxation response to ACh (b). Cumulative concentration response curve to SNP (c). Values are displayed as mean ± SD and represent *n* = 5–8 mice. Statistical analysis was done using two-way repeated measures ANOVA followed by Bonferroni posttest. ^*∗*^ *p* < 0.05 versus IA, ^#^ *p* < 0.05 versus IH, and ^$^ *p* < 0.05 versus IADB. ACh = acetylcholine, SNP = sodium nitroprusside, and PE = phenylephrine.

Endothelial dependent vasodilation to ACh was reduced by CIH (*E*_max_ (%): IA 95.1 ± 3.3% versus IH 67.2 ± 6.5%, *p* < 0.05). The loss of dilation caused by CIH alone was similar to that in IADB (*E*_max_ (%): 60.4 ± 6.1%, *p* > 0.05). The reduction in maximal vasodilatory response to ACh was greatest in the IHDB group (*E*_max_ (%): 33.3 ± 4.7%) which was significantly lower than in IA, IH, and IADB (*p* < 0.05) as shown in Figures 1(a) and 1(b). The EC_50_ (half-maximal effective concentration) for ACh was similar in all groups (log EC_50_ (M): IA: −7.2 ± 0.1, IH: −7.0 ± 0.2, IADB: −7.2 ± 0.1, and IHDB: −7.0 ± 0.1, *p* = NS). Endothelium-independent relaxation to sodium nitroprusside (SNP) was similar in all groups (Figure 1(c)).

### 3.2. Basal NO Production

**Figure 2:**
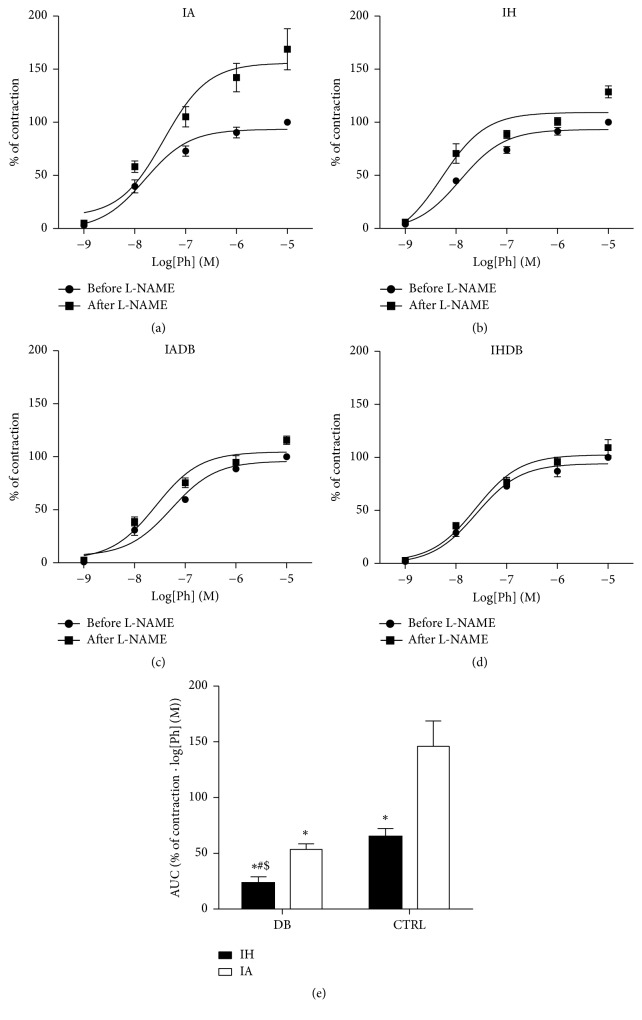
Effect of CIH on basal NO production in diabetic and nondiabetic mice. Cumulative concentration response curves to PE before and after adding L-NAME (a, b, c, d). AUC calculated for the contraction response to PE after adding L-NAME (e). Values are displayed as mean ± SD and represent *n* = 5–8 mice. Statistical analysis was done using two-way repeated measures ANOVA followed by Bonferroni posttest. ^*∗*^ *p* < 0.05 versus IA, ^#^ *p* < 0.05 versus IH, and ^$^ *p* < 0.05 versus IADB. ACh = acetylcholine, AUC = area under the curve, L-NAME = N_*ω*_-nitro-l-arginine methyl ester, and PE = phenylephrine.

Blood vessels maintain a vasodilatory tone through the basal production of NO at rest. The maximal contraction to PE in the IA group was increased after incubation with L-NAME (*E*_max_ (%): IA: 168.6 ± 19.4; see Figure 2(a)). The changes in *E*_max_ for PE in the remaining groups were as follows: *E*_max_ (%): IH: 128.6 ± 3.9, IADB: 115.7 ± 5.5, and IHDB 109.3 ± 7.6 (Figures 2(b), 2(c), and 2(d)). Basal NO production (Figure 2(e)) was significantly higher in IA group (AUC: IA: 146.0 ± 10) when compared to IH and IADB groups (AUC: IH: 65.0 ± 3.2, IADB: 53.5 ± 205, *p* < 0.05). Moreover, basal NO production was the lowest in the IHDB group (AUC: IHDB: 23.2 ± 2.8, *p* < 0.05).

### 3.3. Oxidative Stress, Inflammation, and ADMA in Diabetic Mice Subjected to CIH

**Figure 3:**
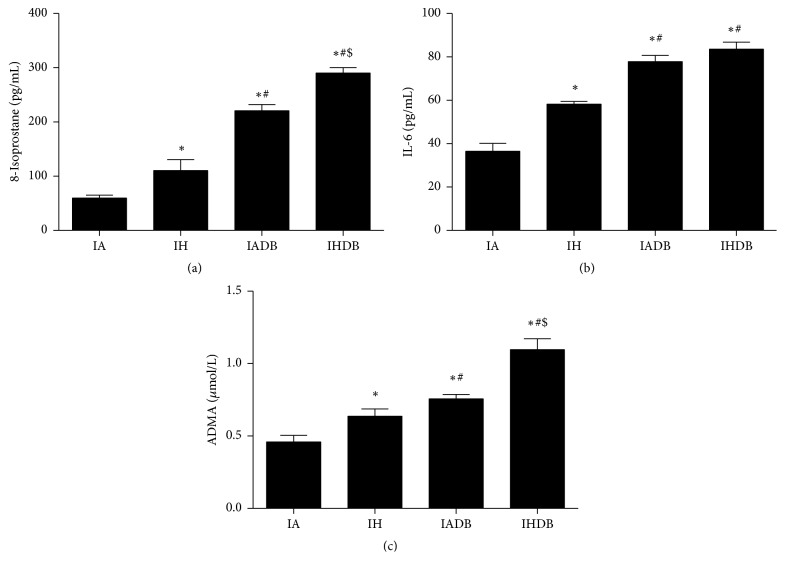
Plasma oxidative stress and inflammation markers with ADMA. Plasma levels of 8-isoprostane (a). Plasma levels of IL-6 (b). Plasma levels of ADMA (c). Values are displayed as mean ± SD and represent *n* = 5 mice. Statistical analysis was done using two-way repeated measures ANOVA followed by Bonferroni posttest. ^*∗*^ *p* < 0.05 versus IA, ^#^ *p* < 0.05 versus IH, and ^$^ *p* < 0.05 versus IADB. IL-6 = interleukin-6. ADMA = asymmetric dimethylarginine.

Levels of plasma 8-isoprostane, a marker of oxidative stress, were significantly increased in both IH and IADB groups (110.1 ± 20.4, 220.6 ± 11.4 pg/mL) compared to IA group (59.6 ± 5.6 pg/mL, *p* < 0.05) and highest in IHDB group (291.2 ± 10.2 pg/mL, *p* < 0.05) (Figure 3(a)). Plasma levels of IL-6, a marker of inflammation, were significantly increased in IH and IADB groups (57.4 ± 2.6, 78.5 ± 2.8 pg/mL) when compared to IA group (36.5 ± 3.6 pg/mL, *p* < 0.05), with levels in IHDB group (83.5 ± 3.1 pg/mL) being greater than in IH and IA (*p* < 0.05) groups but not different from IADB group (*p* = NS) (Figure 3(b)). The plasma levels of ADMA were increased in IH and IADB groups (0.63 ± 0.04, 0.75 ± 0.03 *μ*mol/L) compared to IA group (0.46 ± 0.05 *μ*mol/L, *p* < 0.05) (Figure 3(c)). The increase in ADMA levels in IHDB (1.09 ± 0.05 *μ*mol/L) was significantly higher than in the other groups (*p* < 0.05).

### 3.4. eNOS Uncoupling in Diabetic Mice Subjected to CIH

**Figure 4:**
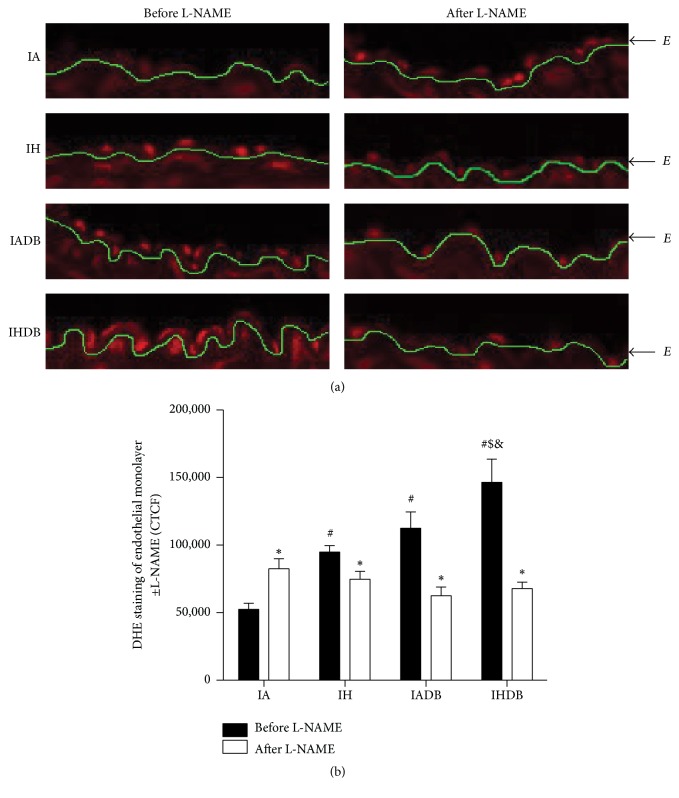
Uncoupled eNOS in the endothelium. Representative images of DHE staining of endothelial monolayer with and without incubation with L-NAME (20x magnification) (a). Quantification of fluorescence levels using CTCF (b). Values are displayed as mean ± SD and represent *n* = 5-6 mice. Statistical analysis was done using Student's *t*-test which was used as a statistical test within groups before and after L-NAME. ^*∗*^ *p* < 0.05 before and after L-NAME. Two-way repeated measures ANOVA followed by Bonferroni posttest was used for comparison between groups after L-NAME. ^#^ *p* < 0.05 versus IA, ^$^ *p* < 0.05 versus IH, and ^&^ *p* < 0.05 versus IADB. CTCF: corrected total cell fluorescence, DHE = dihydroethidium, and L-NAME = N_*ω*_-nitro-l-arginine methyl ester.

 Increased eNOS uncoupling characterizes endothelial dysfunction as it hinders the production of NO. Uncoupling of eNOS was measured by using L-NAME to block eNOS and then staining the aortic segments with DHE (Figure 4). Increased fluorescence in the control group (IA) after L-NAME incubation is due to the prevention of superoxide scavenging by NO (CTCF before L-NAME: 52519 ± 4509 versus after L-NAME: 82473 ± 7408, *p* < 0.05). In contrast, decreased fluorescence in IH, IADB, and IHDB groups after incubation with L-NAME indicated that eNOS is uncoupled and a source of superoxide. The highest fluorescence before adding L-NAME occurred in IHDB group (CTCF: 146502 ± 17183) when compared to both IH and IADB groups (CTCF: IH: 94884 ± 4617, IADB: 112484 ± 12125, *p* < 0.05).

## 4. Discussion

We demonstrate that CIH further exacerbates an already compromised endothelial function in diabetic mice as indicated by the deterioration of ACh-dependent vasodilation and basal NO in diabetic mice exposed to CIH. Our study also demonstrates that CIH increases (1) oxidative stress that was potentiated by diabetes, (2) inflammation that was not significantly worsened by diabetes, (3) ADMA that was aggravated by diabetes, and (4) eNOS uncoupling which was exacerbated by diabetes.

Both CIH and diabetes cause endothelial dysfunction but this effect was more marked in combination. The effects of OSA on endothelial function in diabetic patients have not been reported. Emerging evidence indicates that the risk of developing diabetes associated with sleep disturbances, such as OSA, is comparable to traditional risk factors [22]. Moreover, many diabetic patients are at risk of developing OSA [23]. Our study aimed to assess endothelial function when both conditions are combined, as these are independent risk factors for CVD. Our findings on endothelial dysfunction caused by CIH are supported by other studies in the mouse aorta [24, 25], rat intrahepatic circulation [26], and cerebral and skeletal muscle resistance arteries [14]. We report that the magnitude of endothelial dysfunction caused by CIH is comparable to that caused by diabetes and may be related to the intensity and duration of CIH applied which differs in other studies [11, 27, 28].

We explored some of the potential mechanisms involved in endothelial dysfunction. Both oxidative stress and inflammation are key underlying mechanisms associated with CVD in OSA and diabetes [29–31]. Oxidative stress and inflammation lead to endothelial dysfunction and atherosclerosis [32]. Our results show that plasma 8-isoprostane levels are increased in mice subjected to CIH, as also reported by others [33, 34]. Increased oxidative stress leads to endothelial dysfunction by decreasing NO bioavailability and eNOS uncoupling and increasing peroxynitrite production in OSA [29]. Levels of 8-isoprostane in* db/db* mice were double that measured in nondiabetic mice exposed only to CIH. The differences in oxidative stress levels may be related to different pathways and sources of ROS [30, 31]. The magnitude of oxidative stress is related to the severity of CIH, animal species, and organs studied [35, 36]. The greatest increase in 8-isoprostane occurred in* db/db* mice exposed to CIH, suggesting that CIH exacerbates oxidative stress in diabetes to further deteriorate endothelial dysfunction.

The major inflammatory pathway in OSA is initiated through the activation of the nuclear factor-kappaB (NF-*κ*B). This transcription factor is responsible for the expression of inflammatory cytokines such as IL-6 and adhesion molecules such as intracellular adhesion molecule-1 (ICAM-1) [37]. It has been previously demonstrated that CIH induces inflammation through increased levels of IL-6 [38, 39]. Inflammation that occurs in diabetes is an important contributor of vascular dysfunction in* db/db* mice [18, 40]. Our data demonstrates that the increased levels of IL-6 in* db/db* mice were not further exacerbated by CIH, likely due to the already high levels of inflammation already present in diabetic mice.

The function of eNOS is compromised in diseases such as diabetes and hypertension, where eNOS produces superoxide anion instead of NO by a process termed “eNOS uncoupling” [41]. Tetrahydrobiopterin (BH_4_) is a cofactor essential for NO production; oxidative stress leads to its oxidation and eNOS uncoupling [42]. Supplementation with BH_4_ reverses endothelial dysfunction in OSA patients [43]. We evaluated eNOS uncoupling in the endothelial layer of aortic sections by measuring the fluorescence of the superoxide-sensitive dye (DHE) with or without incubation with L-NAME (eNOS inhibitor). Increased fluorescence in control mice after L-NAME incubation (compared to no incubation) indicates decreased NO availability for interaction with superoxide anion. On the other hand, decreased fluorescence after L-NAME incubation indicates blockage of uncoupled eNOS due to lower amounts of superoxide anion, as seen in both CIH and diabetic groups. Furthermore, eNOS uncoupling was more prominent in* db/db* mice exposed to CIH. Although aortic DHE staining is nonspecific (e.g., can also detect hydrogen peroxide) as shown by Oelze et al. in diabetic rats [44], the decreased signal in DHE after L-NAME incubation suggests inhibiting uncoupled eNOS which mainly produces superoxide anion [45]. This data suggests that increased eNOS uncoupling could be related to increased oxidative stress and can partially explain the exacerbated endothelial dysfunction seen in diabetic mice subjected to CIH.

ADMA is a natural endogenous eNOS inhibitor, which competes with L-arginine to reduce endothelial NO production and cause eNOS uncoupling [46, 47]. Oxidative stress can increase ADMA levels by oxidizing and deactivating the enzyme responsible for ADMA elimination (dimethylarginine dimethylaminohydrolase (DDAH)) [43]. Our findings reveal that ADMA levels are increased by CIH and diabetes, with ADMA levels being greater in* db/db* mice exposed to CIH. This suggests the involvement of ADMA as a contributor to endothelial dysfunction.

In summary, endothelial dysfunction was exacerbated in* db/db* mice when subjected to CIH. Increased eNOS uncoupling, 8-isoprostane, and ADMA levels potentially caused by oxidative stress may explain the detrimental effects of CIH in diabetic mice. These data suggest that it would be important to evaluate oxidative stress and endothelial function in diabetic patients who are diagnosed with OSA and that patients with concomitant OSA and diabetes may represent a group of patients at particularly high risk of CVD. A better understanding of the interaction between OSA and diabetes may facilitate tailored treatments for this population and improve cardiovascular outcomes.

## Abbreviations Used

ACh: Acetylcholine
AUC: Area under the curve
BH_4_: Tetrahydrobiopterin
CIH: Chronic intermittent hypoxia
DHE: Dihydroethidium
ELISA: Enzyme-linked immunosorbent assay
eNOS: Endothelial nitric oxide synthase
FIO_2_: Fraction of oxygen inspired
IA: Intermittent air nondiabetic
IADB: Intermittent air diabetic
ICAM-1: Intracellular adhesion molecule-1
IH: Intermittent hypoxia nondiabetic
IHDB: Intermittent hypoxia diabetic
IL-6: Interleukin-6
L-NAME: N_*ω*_-Nitro-L-arginine methyl ester
NF-*κ*B: Nuclear factor-kappaB
NO: Nitric oxide
PE: Phenylephrine
ROS: Reactive oxygen species
SNP: Sodium nitroprusside.

## Additional Points

*Limitations*. Although our mouse model resembles CIH occurring in OSA patients, the mice do not experience obstruction of the upper airway. Thus, there is no induction of hypercapnia or negative intrathoracic pressure swings that are typically seen in patients with OSA. These physiologic changes may affect endothelial function and cardiovascular outcomes. In addition, the degree of hypoxia and oxyhemoglobin desaturation experienced by the mice in our experiment is more severe than that commonly seen in the majority of patients with OSA. As such, this model would be more applicable to patients with severe OSA. Another limitation is the nonspecificity of DHE staining, which can also detect hydrogen peroxide and thus affect the fluorescence signal. Finally, the diabetic mice we used (*db/db*) suffer from obesity and hyperleptinemia, and these factors may have effects on endothelial function independent of diabetes per se.
